# Inhibitory insula-ACC projections modulate affective but not sensory aspects of neuropathic pain

**DOI:** 10.1186/s13041-023-01052-8

**Published:** 2023-08-21

**Authors:** Heloísa Alonso-Matielo, Zizhen Zhang, Eder Gambeta, Junting Huang, Lina Chen, Gabriel Oliveira de Melo, Camila Squarzoni Dale, Gerald W. Zamponi

**Affiliations:** 1 Department of Clinical Neurosciences, Alberta Children’s Hospital Research Institute and Hotchkiss Brain Institute, Cumming School of Medicine, University of Calgary, Calgary, AB Canada; 2 Department of Anatomy, Institute of Biomedical Sciences of University of São Paulo, Av. Prof. Lineu Prestes, 2415, ICB-III, Cidade Universitária, São Paulo, SP 05508-900 Brazil

**Keywords:** Insular cortex, Anterior cingulate cortex, Neuropathic pain, Neural projection, Optogenetics

## Abstract

The insula and anterior cingulate cortex (ACC) are brain regions that undergo structural and functional reorganization in neuropathic pain states. Here, we aimed to study inhibitory parvalbumin positive (PV+) posterior insula (pIC) to posterior ACC (pACC) projections, and to evaluate the effects of direct optogenetic manipulation of such projections on mechanical nociception and spontaneous ongoing pain in mice with Spared Nerve Injury (SNI). CTB_488_ tract-tracing in male PVCrexAi9 mice revealed a small proportion of PV+ projections from the pIC to the pACC. Electrophysiological analysis confirmed the existence of synaptic inputs into the pACC by pIC GABAergic cells. Optogenetic stimulation of these pathways did not change mechanical nociception, but induced conditioned place preference behavior responses. Our results suggest the presence of inhibitory projections between the pIC and the pACC which are able to selectively modulate affective aspects of neuropathic pain.

**Supplementary Information:**

The online version contains supplementary material available at 10.1186/s13041-023-01052-8.

The pain modulatory system comprises anatomical pathways that include inhibitory circuits originating from cortical areas such as the insula and anterior cingulate cortex (ACC) [1]. The posterior insula (pIC) is involved in the processing and modulation of sensory functions [2] and connects with pain matrix structures such as the ACC [3, 4]. Insula activity is affected by different painful stimuli and altered during neuropathic pain in preclinical models and in patients [5, 6]. The insula contains parvalbumin (PV+) expressing inhibitory interneurons [7] and GABAergic inhibition in the pIC is directly involved in induction of analgesia [6, 8]. Similarly, in the ACC, decreased pain responses and associated aversive behavior are mediated by inhibitory signaling [5, 9]. We thus aimed to examine the existence and role of inhibitory projections from the pIC to the pACC, and to test their role in hypersensivity and affective-motivational behavior of SNI mice. Detailed methodologies are provided in Additional file 1.

We first examined to what extent the insular cortex and pACC are linked by inhibitory connections. CTB_488_ was injected into the pACC region of PV-tdTomato mice, and overlap between CTB_488_ and tdTomato label in slices from the insular cortex visualized and quantified. The green retrotracer-labeled GABAergic cells in the pIC are shown in Fig. 1a, and the correct location of the CTB_488_ injection was verified by examination of pACC slices (Fig. 1b). Quantitative analysis (Fig. 1c) reveals the percentage of tdTomato positive cells that overlap with CTB_488_ fluorescence in the pIC (SHAM: 4.4 ± 4.4%, n = 3; SNI: 45.1 ± 26.3%, n = 4). We also examined the degree of c-Fos activation in GABAergic cells projecting to the pACC in the presence and absence of SNI (Fig. 1d). These data suggest very little tonic activation of these neurons during both conditions, with only around 10–20% of projecting cells being activated after SHAM surgery, and perhaps even less activation after SNI (proportion of c-Fos positive PVCre::tdTomato-CTB_488_ cells: SHAM 16.6 ± 16.6%, n = 3; SNI 0 ± 0, n = 3). Hence, SNI does not appear to induce increased activity in GABAergic pIC to pACC projections.

**Fig. 1:**
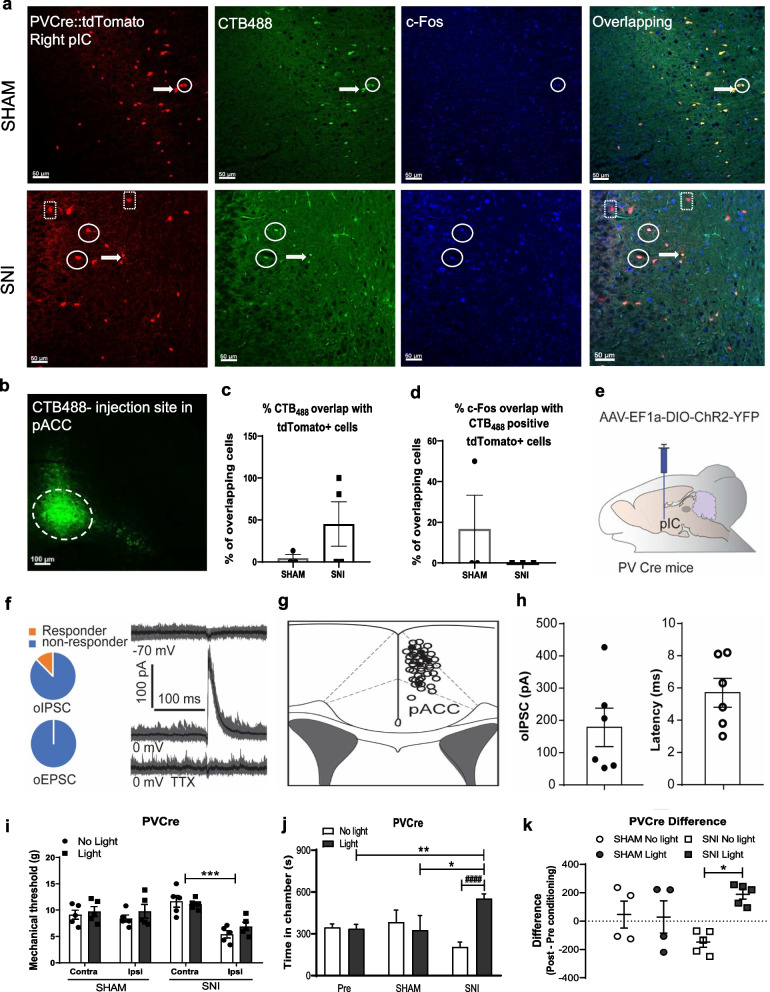
Insula-pACC PV+ projections modulate CPP responses without mechanical threshold effects.** a** Confocal microscopy images from pIC slices from SHAM and SNI operated PV Cre:tdTomato mice subjected to CTB_488_ (green) injection in the pACC (PVCre n = 7) followed by SNI or SHAM procedures a week later. Slices were immunostained for c-Fos activity (shown in blue). White arrows highlight examples of cells in which CTB_488_ and tdTomato label overlap, circles highlight projecting cells that are positive for c-Fos. Dotted squares show only PV+ cells. **b** Verification of injection of CTB_488_ injection sites in the pAAC region. **c** Quantification of the percentage overlap between tdTomato positive cells that are also positive for CTB_488_. **d** Percentage overlap between pIC-pACC projecting GABAergic cells (i.e., cells that are both red and green) and those positive for c-Fos (blue). Data are presented as mean ± S.E.M. **e** Diagram showing that AAV (AAV9-EF1a-DIO-ChR2-eYFP) was injected in the pIC of PV Cre transgenic mice. **f** Voltage clamp recording in brain slices of the pACC. Left: percentage of cells that responded with oIPSCs (12.7%, upper) and with oEPSCs (0%, lower). Right: sample traces showing responses at − 70 mV (no oEPSC) and at 0 mV (with oIPSC) and that TTX blocked the oIPSC response. **g** Location of recording sites in pACC slices. Each recorded neuron in the pACC was mapped onto a brain section of pACC from a mouse brain atlas showing the location of responding neurons (responder, solid dots) and non-responding neurons (non-responder, open dots). **h** Amplitude and latency of oIPSCs from responding neurons. Error bars are S.E.M. **i** Mechanical withdrawal threshold of SHAM and SNI operated PVCre mice with and without optoactivation of ChR2 (Light and No Light-473 nm, pulsed light, 5mW, 40 Hz) and mechanical threshold was accessed using a DPA test with the animal under stimulation. Two-way ANOVA followed by Bonferroni post-test. ***p = 0.0001. **j** Optogenetic stimulation of the GABAergic pIC to pACC pathway was able to induce place preference in SNI mice **(**PVCre SNI (n = 5) or SHAM (n = 4 to 5)). The paradigm consists of one day conditioning, with lights off for 15 min in the morning in one chamber and opto-light stimulation for 15 min in the afternoon in the opposite chamber. Two-way ANOVA followed by Bonferroni post-test, *p = 0.0331 SHAM vs SNI Light paired chamber; **p = 0.0082 Pre (pre-test) vs SNI Light paired chamber; ####p < 0.0001 Light vs No Light SNI group. **k** CPP score (difference of time spent in the conditioning chamber during post- and pre-conditioning); One-way ANOVA followed by Bonferroni post-test, *p = 0.018 SNI No Lght vs Light. All data are presented as mean ± S.E.M

To complement our retro-tracing experiments, we used electrophysiology on pACC slice preps with opto-stimulation of synaptic inputs from the pIC. AAV-EF1a-DIO-ChR2-H134R was injected in the pIC of PVCre transgenic mice to express ChR2 in PV+ neurons (Fig. 1e). Whole cell patch clamp recordings were performed in neurons of the pACC, using blue laser stimulation (10 ms, every 20 s, Laserglow ON) of the input fibers originating from the pIC. Forty-seven cells (7 mice) were randomly chosen for recordings in the pACC (Fig. 1f, g). Among these, 6 cells responded with an oIPSC at 0 mV, but none responded at − 70 mV (Fig. 1f, left). TTX was applied to 3 of the 6 responding cells, leading to complete loss of the oIPSC, suggesting that the oIPSC responses are action potential dependent (Fig. 1f, right). Figure 1h illustrates the amplitudes of the optically evoked IPSCs and their latencies. Altogether, these data indicate that the pACC receives sparse but functional innervation from pIC PV+ GABA cells and confirm the confocal microscopy data. We note that our approach does not allow us to discern whether the recorded cells were GABAergic or glutamatergic.

For behavior characterization, AAV9-Ef1α-DIO-ChR2-EFYP was injected in the pIC of PVCre mice followed by the implantation of a fiber optic cannula in the pACC. Histological slices were used to confirm stereotaxic coordinates for both sites of injection and implantation (not shown). PVCre SHAM and SNI mice were subjected to optostimulation with a blue laser for selective ChR2 activation in PV+ projections from the pIC to the pACC. There was no effect of optostimulation on mechanical withdrawal thresholds in SHAM mice (No Light: 8.4 ± 0. 7 g; Light: 9.8 ± 1.3 g; n = 5) (Fig. 1i). SNI mice exhibited ipsilateral mechanical hypersensitivity that was not reversed by optostimulation (No Light: 5.4 ± 0.7 g; Light: 6.9 ± 0.8 g; n = 5). These data indicate that exogenous optogenetic activation of GABAergic pIC to pACC projections does not affect sensory aspects of neuropathic pain. The effect of optostimulation on Conditioned Place Preference (CPP) was then evaluated. In Fig. 1j, PVCre SNI mice exhibited place preference when conditioned with blue light (SNI: Light 554.0 ± 32.2 s; n = 5; No Light 205.6 ± 34.6 s, n = 5 ####p < 0.0001). The SHAM group was not affected. These data are also represented in Fig. 1k in the form of difference scores (SNI Light 188.0 ± 32.8 s; SNI No light = − 148.4 ± 36.2 s; n = 5, *p = 0.0182). Collectively, these data suggest that activation of PV + projections from the pIC to the pACC participate in affective aspects of neuropathic pain.

Several interconnected brain regions have been implicated in neuropathic pain, including the prefrontal cortex [10, 11], amygdala, PAG and thalamus [1]. The insula and the ACC are two major areas that appear to be involved in pathological pain [12], and most importantly in the control of sensitive-discriminative and affective-motivational pain aspects [13, 14]. Previous findings have associated decreased insular GABA levels with hyperalgesic behavior in animals [13], suggesting that inhibitory tone in insula may be a causal contributor to the development of chronic pain states. Indeed, inhibitory signaling in both the insular cortex and ACC is responsible for the induction of analgesia [5, 12]. Our observation that optostimulation of the GABAergic projections resulted in altered CPP supports that these projections are physiologically relevant. Sensory components of neuropathic pain were not affected suggesting that connections between the pIC and the pACC discriminate between these modalities. This may fit with the notion that the pACC can integrate sensory and affective aspects of pain [14]. Yet a previous study revealed that a projection from the mid-cingulate cortex to the insula does control nociceptive hypersensitivity, suggesting that the pIC may perhaps modulate affective and sensory components of chronic pain depending on which pathway is activated [15].

Only a small portion of pIC to pACC projections were activated under basal conditions, and the proportion of activated cells appeared even lower after SNI surgery. c-Fos labeling has limitations, including a lack of quantitative output of neuronal activity, and subtle activity alterations within a specific subset of already active neurons due to SNI may not have been resolved. Our sample size was, however, limited, and thus putative activity differences between SHAM and SNI surgery may not have been fully resolved.

In summary, our results support the idea that inhibitory pathways between the pIC and pACC can modulate affective-motivational aspects of pain, highlighting these areas as potential targets for the management of neuropathic pain.

### Supplementary Information

**Additional file 1. Materials and Methods**

## Data Availability

All data generated or analysed during this study are included in this published article, data will be made available upon reasonable request.

## Abbreviations

CPP: Conditioned place preference
pIC: Posterior insular cortex
pACC: Posterior anterior cingulate cortex
SNI: Spared nerve injury
PV: Parvalbumin
PAG: Periaqueductal grey
EYFP: Enhanced yellow fluorescence protein
GABA: Gamma aminobutyric acid
