# Supplementary material for: Inhibitory insula-ACC projections modulate affective but not sensory aspects of neuropathic pain
Source: Mol Brain. 2023 Aug 21;16:64. doi: 10.1186/s13041-023-01052-8 (PMC10440912; doi:10.1186/s13041-023-01052-8)

Inhibitory insula-ACC projections modulate affective but not sensory aspects of neuropathic pain

Heloísa Alonso-Matielo[a,b], Zizhen Zhang[a], Eder Gambeta[a], Junting Huang[a], Lina Chen[a] , Gabriel Oliveira de Melo[b], Camila Squarzoni Dale[b], Gerald W. Zamponi[a]

[a] Department of Clinical Neurosciences, Alberta Children's Hospital Research Institute and Hotchkiss Brain Institute, Cumming School of Medicine, University of Calgary, Calgary, Canada.
[b] Department of Anatomy, Institute of Biomedical Sciences of University of São Paulo – Av. Prof. Lineu Prestes, 2415, ICB-III, Cidade Universitária, 05508-900 – São Paulo, SP, Brazil.

Additional Supplemental Information

Materials and methods

Animals

6-8 week-old male mice from different lineages were used throughout the study. All protocols were approved by the Animal Care Committee at University of Calgary, Canada (AC16-0191), and experiments were carried out according to the guidelines of the National Institutes of Health (NIH) to minimize all animal stress and suffering. Cre transgenic mice PV-cre: B6; 129P2-Pvalb$^{tm1\ (cre)\ Arbr\ /\ J}$ (Jackson Laboratories) were used for all optogenetics experiments. They were crossed with B6.Cg-Gt (ROSA) 26Sor$^{tm9\ (CAG\text{-}tdTomato)\ Hze\ 369\ /\ J}$ for labeling PV+ (PVCrexAi9) cells. Animals were maintained in a room with controlled temperature (23°C±1°C) and 12h/12h light/dark cycle, with food and water *ad libitum*.

Experimental design

Transgenic Cre mice PV-cre: B6; 129P2-Pvalb$^{tm1\ (cre)\ Arbr\ /\ J}$ (Jackson Laboratories, Maine, USA) were first crossed with mice carrying the tdTomato Ai9 reporter gene: $^{B6.Cg\text{-}Gt(ROSA)26Sortm9(CAG\text{-}tdTomato)Hze369/J}$ for labeling of parvalbumin (PVCrexAi9) neurons.

PVCrexAi9 mice received an injection of the retrograde neuronal tracer cholera toxin subunit B ALEXA-488 (CTB_{488} 0.1% weight / volume; 400- 450 nl / animal- Thermo Fisher Scientific, Massachusetts, USA) in the right pACC and the Spared Nerve Injury (SNI) was performed on the left sciatic nerve a week later. 14 days after SNI, animals were subjected to a mechanical sensitivity test using Dynamic Plantar Aesthesiometer (DPA) and then euthanized through transcardiac perfusion. Brain samples were collected and histological samples were used to observe cell bodies within the pIC using confocal microscopy (Leica Microsystems, Wetzlar, Germany). Slides were also subjected to immunofluorescence for c-Fos identification of neuronal activation (Figure 1a).

We also used PV-cre: B6; 129P2-Pvalb$^{tm1\ (cre)\ Arbr\ /\ J}$ mice (Jackson Laboratories, Maine, USA) (males, 6-8 weeks old) which were injected with AAV vectors for selective channelrhodopsin (ChR2) expression in neurons. Two weeks later, a fiber optic cannula was implanted in the right pACC. Four weeks following this procedure, mice underwent SNI or SHAM surgery in the left sciatic nerve. 8 weeks after virus injection, mice were subjected to opto-light stimulation with blue light with a power of 5mW measured at the fiber tip (S130C power sensor, Thorlabs, Nova Jersey, USA). 40Hz pulsed light was used for stimulation of PV+ cells, and mice were subjected to the DPA and CPP tests. Animals were euthanized and brain samples collected after transcardiac perfusion

with 0.1M PBS and PFA4%, and samples were used to confirm AAV expression and cannula implantation. A schematic representation of the protocol is shown in Figure S1b.

Spared Nerve Injury model

The SNI model was performed according to previous reports [1]. The left sciatic nerve was dissected at the level of trifurcation and a tight knot was made on tibial and common peroneal nerves with a silk suture line (6-0) with 1mm of the nerve removed distal to the ligature. The sural nerve was kept intact. The muscles and the skin were sutured separately with silk (6-0) and Vicryl suture line (4-0), respectively. SHAM operated mice were subjected to the same surgical procedure, without nerve lesioning.

CTB_{488} tract-tracing

PVCrexAi9 mice were anesthetized with isoflurane (5% for induction and 2.5% for maintenance) and subjected to stereotaxic surgery to allow placement of a glass capillary to inject CTB_{488} (CTB_{488}, 0.1% weight / volume; 400- 450 nl / animal- Thermo Fisher Scientific, Nova Jersey, USA, 100 nl/ minute) in the right pACC (coordinates 1.055mm anterior to bregma, 0.255 right to the midline, and 1.622mm depth) for retrograde labeling of pACC projecting neurons. The glass capillary was kept in position for 5 min, then raised 100 µm and kept in position for more 10 min to allow solution diffusion. To retrogradely trace pIC-pACC long-range projections CTB_{488} was allowed 21 days for sufficient neuronal transport and labeling. Five images of labeled cells from three to four animals per group injected were obtained using confocal microscopy (Leica Microsystems, Wetzlar, Germany), and ImageJ software used to cell counting and analysis.

Immunofluorescence for c-Fos labeling

Brain samples from PVCrexAi9 SNI or SHAM mice were collected 90 min following paw stimulation by means of the DPA test, through transcardiac perfusion with 0.1 M PBS and PFA 4%. Samples were stored in PFA 4% overnight then transferred to 30% sucrose solution until cryosectioning. Immunofluorescence for c-Fos was performed to confirm that PV+ GABAergic cells next to cells optoactivated by ChR2 did not already present c-Fos activation. Imunofluorescence was performed on slides prepared with coronal sections (30µm slice thickness) as follows: slides were washed in 0.1 M PB three times then incubated in blocking solution (Triton X-100 0.3% and 5% NGS) for 90 min at room temperature, and incubated overnight with Fos-specific primary antibody diluted in Triton X-100 0.3% and 5% NGS (rabbit c-Fos sc-52 ab190289; 1:1000; Santa Cruz Biotechnology, Inc, USA). Slides were washed in 0.1 M PBS and incubated at room

temperature for 150 min with secondary antibody Alexa Fluor 633 goat anti-rabbit (1:600, goat anti-rabbit A-21070 Invitrogen, Thermo Fisher Scientific, USA), shown in blue in confocal microscopy images). The slides were washed in 0.1 M PBS and mounted on cover slips with Fluoromount/Plus^{TM} Plus anti-fading mounting medium (Cedarlane, Canada). Immunoreactivity was observed using a 10×0.4 NA and a 20×0.75 NA objective lens on a Leica TCS SP8 confocal microscope, and images were captured in LAS X Life Science Leica software (Leica Microsystems, Wetzlar, Germany). For each mouse, three slices were captured, images analyzed using ImageJ software and the mean ± s.e.m of the number of c-Fos- immunolabeled nuclei was calculated for SNI and SHAM groups.

AAV injection and virus expression

PV-cre: B6; 129P2-Pvalb$^{tm1\ (cre)\ Arbr\ /\ J}$ (Jackson Laboratories, USA) mice were subjected to stereotaxic injection of AAV vectors for selective expression of ChR_2 in neurons: pAAV5-EF1a-DIO-hChR2 (H134R)-EYFP (12x10^{12} particles / ml; 400-450 nl / animal; Addgene); or pAAV9-EF1a-DIO-hChR2 (H134R)- EYFP (12x10^{13} particles / ml; 100-200 nl / animal; Addgene), injected into the right pIC (0.5 mm posterior to the bregma, 0.375 mm laterality and 4.1 mm depth). The skull was exposed, cleaned with saline 0.9% and a hole was drilled on the skull according to the pIC coordinates above. A glass capillary was used to inject the virus at 50 nl bolus /30 s. The capillary remained in position for 5 min, was raised 100 µm and kept in the position for more 10 min to allow solution diffusion. Skin was sutured with Vicryl suture line (4-0) and Polysporin (antibiotic and analgesic combination) was applied topically.

Eight weeks later, 90 min following the DPA test brain samples from PVCre mice that had been subjected to SNI or SHAM surgeries and optostimulation were collected through transcardiac perfusion with 0.1 M PBS and PFA 4%. Samples were stored in PFA 4% overnight then transferred to 30% sucrose solution until cryosectioning (30 µm) and mounted on microscope slides. Slides were washed in 0.1 M PBS and mounted on coverslips with Fluoromount/Plus^{TM} Plus anti-fading mounting medium (Cedarlane, Canada). Positive AAV-expressing cells (pAAV9-EF1a-DIO-hChR2 (H134R)- eYFP) were observed using a 10×0.4 NA and a 20×0.75 NA objective lens on a Leica TCS SP8 confocal microscope and images scanned in LAS X Life Science Leica software (Leica Microsystems, Wetzlar, Germany).

Fiber Optic cannula implantation

6-8 week old male PVCre mice were anesthetized with isoflurane (5% for induction and 2.5% for maintenance) and subjected to stereotaxic implantation of a fiber optic cannula (2.5 mm ceramic

ferrule; 2.0 mm length; 200 µm core diameter and 0.39 numerical aperture (NA), Thorlabs, USA). In this surgery, the skull was exposed and cleaned with saline solution 0.9% and hydrogen peroxide and a small hole was drilled right above the target site for implanting the fiber optic cannula. PVCre mice were implanted in the right pACC (coordinates: 1.055 mm anterior to bregma; 0.255 mm right to the midline and 1.522 mm of depth). The surgery was performed 14 days after virus injection. C&B Metabond adhesive (Parkel Inc, USA) and a plastic cap were used to fix and protect the implant. Skin was sutured using a Vicryl suture line (4-0) and Polysporin (antibiotic and analgesic combination) was applied above the skin surface.

In vivo optogenetics stimulation

Eight weeks after AAV injection animals were habituated for 30 min before any manipulation. Animals were then anesthetized with 2% isoflurane to allow connecting the fiber optic cannula to a patch cable with a 2.5 mm zirconia sleeve and a rotatory joint coupled to the laser generator. After recovery from anesthesia, ChR2 expressing neurons were stimulated with DPSS lasers at 473 nm blue light (Laserglow Technologies, Ontario,Canada) with 5mW of power light measured at the fiber tip (S130C power sensor, Thorlabs, USA). Blue light (40 Hz, 10ms pulse) was used for stimulation of PV+ cells (PVCre mice), and mimicking gamma frequency in behavioral experiments [1]. Animals were stimulated for 3 min before starting behavioral assessment with continuous stimulation throughout the testing period.

Mechanical nociception evaluation

Mechanical nociception was evaluated using a Digital Plantar Aesthesiometer (DPA) test. Animals were placed individually in an enclosed area on top of a wire mesh floor and the aesthesiometer was positioned directly under the plantar surface of the hind paw for a mechanical pressure testing, evaluated in grams (g), until the withdrawal response of the mice. Each paw (ipsi and contralateral to SNI surgery) was tested three times, with an interval of 10 seconds. Mice were tested in a baseline measurement, before opto-light stimulation (No Light) and after optostimulation (Light).

Conditioned Place Preference (CPP) paradigm

The CPP test was performed as previously described by Zhang et al [1]. The CPP box consists of two conditioning chambers with distinct visual and tactile cues (vertically striped or dotted walls and smooth or meshed floor, respectively), connected by a middle neutral chamber. The protocol started two weeks after the SNI or SHAM surgeries. On day 1 (pre-conditioning), a patch cable was attached to the implanted cannula and the mouse was placed individually in the middle

chamber with free access to both main chambers for 15 minutes. The time (in seconds) spent in each chamber was recorded and animals that spent more than 70% or less than 30% in one of the conditioning chambers were excluded from the analysis. A counterbalance for the chambers was performed to avoid experimental bias. In the following day (day 2 - conditioning), during the morning animals were conditioned in one chamber with no opto-stimulation (No light) for 15 minutes, and 4 hours later (i.e. in the afternoon), animals were conditioned in the opposite chamber with opto-stimulation (Light) for 15 minutes. On day 3 (post-conditioning), mice were treatment-free (no opto-stimulation) and were allowed to freely access all chambers for 15 min and the time spent in each conditioning chamber was recorded. The total time spent in each chamber and the CPP score were calculated as the time spent in the light ON conditioned chamber on the testing day minus the time spent in the same chamber on the preconditioning day (Pre).

Electrophysiology

Acute coronal forebrain slices (260 μm) containing the pACC were obtained from PV transgenic mice. Briefly, coronal sections were cut using a vibratome (Leica VT1200S, Leica Biosystems) in an ice cold NMDG-based solution. Brain slices were first incubated at 33.5°C for 50 min, then transferred to room temperature in normal external solution for at least 1hr before recording. Whole cell patch clamp recordings were performed using a MultiClamp 700B and Digidata 1440A (Molecular Devices). Normal external solution contained: 120 mM NaCl, 1.25 mM NaH2PO4, 26 mM NaHCO3, 25 mM Glucose, 2.5 mM $CaCl_2$, 2.5 mM KCl, and 1.3 mM MgSO4-7H2O. The intracellular solution for voltage clamp was Cs-Methanesulfonate based and contained: 130 mM Cs Methanesulfonate, 4 mM CsCl, 2 mM EGTA, 4 mM Mg-ATP, 0.3 mM Na-GTP, 10 mM HEPES, and 5 mM QX-314. For blue laser evoked excitatory/inhibitory postsynaptic current (oEPSC/oIPSC) recordings in the pACC, Vh was held at – 70 mV (calculated Cl^- reversal potential) and 0 mV (reversal potential for EPSC), respectively. TTX (1 μM) was used to block action potential based synaptic transmission. Transgenic mice injected with AAV in the pIC were allowed to express ChR2 in the terminal area for at least 8-10 weeks before brain slice recording. DPSS blue lasers (Laserglow Technologies, Ontario) was used to illuminate brain slices for recordings (473 nm, 10 ms pulse every 20 s).

Statistical analysis

Results are presented as mean ± standard error of the mean (s.e.m). Statistical analyses of all data were generated using GraphPad Prism, version 8 (GraphPad Software Inc., San Diego, CA, USA). Analysis of Variance (ANOVA) followed by Bonferroni's test was used for parametric data and

comparisons of more than two groups. Paired or unpaired parametric t-test were used for the analysis between two groups. In all cases, $p < 0.05$ is considered statistically significant.

Reference

1. Zhang Z, Gadotti VM, Chen L, Souza IA, Stemkowski PL, Zamponi GW. Role of Prelimbic GABAergic Circuits in Sensory and Emotional Aspects of Neuropathic Pain. Cell Rep. 2015;12:752–9.

Figure S1. Study design. a. Schematic representations for the CTB_{488} tract-tracing experimental protocol in PVCrexAi9 mice subjecred to SNI or SHAM surgeries (times lines in days). **b.** Protocol (in weeks) for the the surgical procedures and behavior assessment performed in mice for *in vivo* optogenetics.

Figure S1

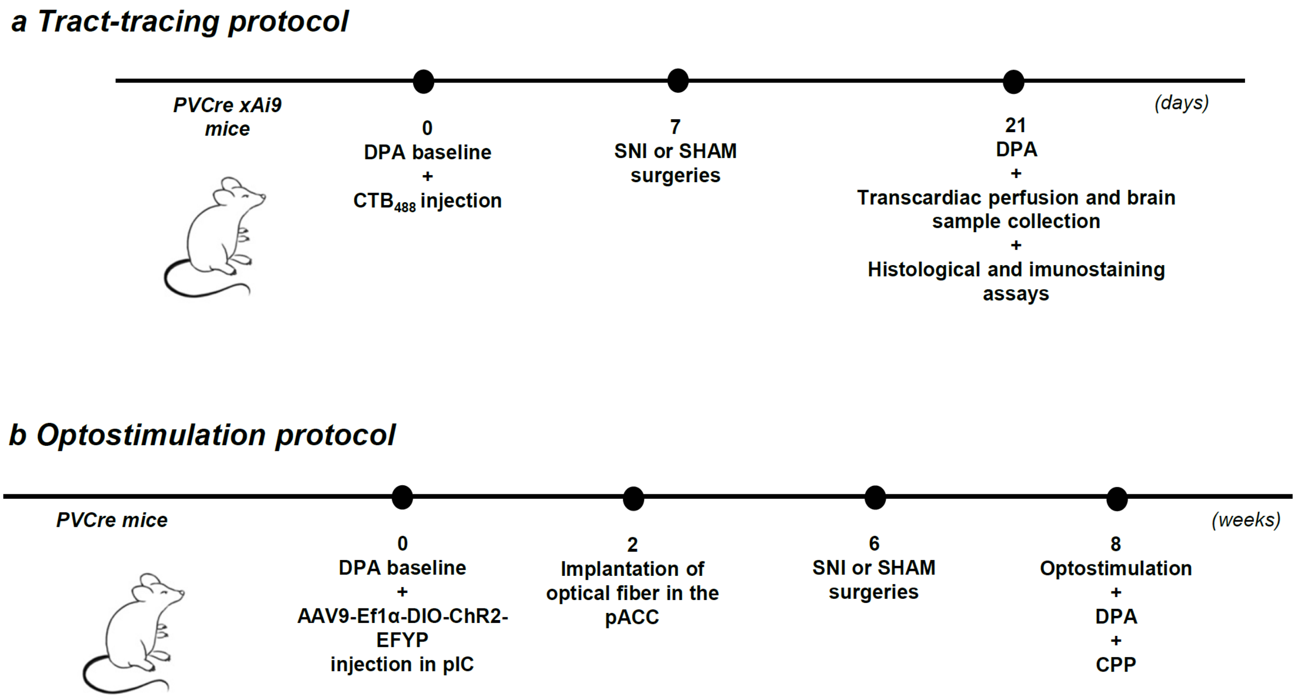

Supplement: Supplementary file 1 — Additional file 1. Materials and Methods [file 13041_2023_1052_MOESM1_ESM.pdf]
